# Genistein upregulates LDLR levels via JNK-mediated activation of SREBP-2

**DOI:** 10.3402/fnr.v60.31120

**Published:** 2016-05-20

**Authors:** Medicia Kartawijaya, Hye Won Han, Yunhye Kim, Seung-Min Lee

**Affiliations:** Department of Food and Nutrition, College of Human Ecology, Yonsei University, Seoul, South Korea

**Keywords:** genistein, JNK, LDL receptor, cholesterol, SREBP-2

## Abstract

**Background:**

Genistein has been proved *in vitro* and *in vivo* to lower LDLR level. It is also widely consumed and implicated for its anti-atherogenic effects. However, the molecular mechanism by which genistein lowers the LDL level is still unknown.

**Objective:**

To understand the anti-atherogenic molecular mechanism of action, genistein was investigated for its impact on the expression of LDLR, the receptor for LDL cholesterol, and related signaling pathways in a human hepatoma cell line.

**Design:**

HepG2 cell was used for the experiments. Genistein with different concentrations was diluted in media and was incubated for 24 h or more as indicated. Protein levels were measured by western blotting, and mRNA expression was detected by RT-qPCR. Chromatin immunoprecipitation assay (CHIP) assay was used to determine protein binding levels, and luciferase assay was used to measure promoter activity.

**Result:**

Genistein increased the mRNA and protein levels of LDLR in a time-dependent manner. Genistein increased the transcriptional activity of the LDLR promoter containing the reporter gene (pLDLR-luc, −805 to +50). But the sterol regulatory element deletion mutant construct failed to be activated by genistein. Genistein increased the nuclear fraction of SREBP-2 and the DNA-binding activity of SREBP-2 to LDLR promoter, as assessed by CHIP. The genistein-phosphorylated JNK inhibitor (SP600126) abolished the genistein-stimulated levels of LDLR and the nuclear SREBP-2. The addition of cholesterol up to 5 µg/mL for 24 h did not affect the effect of genistein on LDLR protein expression. Even the addition of 40 µM genistein increased the cholesterol uptake by more than 10% in the human hepatoma cell line.

**Conclusion:**

Our data support the idea that genistein may have anti-atherogenic effects by activating JNK signals and SREBP-2 processing, which is followed by the upregulation of LDLR.

Hypercholesterolemia is a strong risk factor for the development of atherosclerosis, a hallmark of cardiovascular disorders (1). Familial hypercholesterolemia suggests that the hepatic expression of LDLR, a receptor for LDL cholesterol, is crucial for the regulation of blood cholesterol levels (2, 3). Extensive studies indicate that statins improve lipid profiles by inhibiting the activity of HMGCR, a rate-limiting enzyme of cholesterol synthesis, thereby increasing the expression of LDLR (4). Even so, statins only reduce cardiovascular risk by about 20 to 40% (5). Diet alone successfully lowers blood LDL cholesterol to a degree comparable with statin treatment (6). Combination therapy using statins and a dietary regime may further reduce blood cholesterol levels (5).

Sterol regulatory element-binding protein 2 (SREBP-2) is a key transcription factor in cholesterol metabolism. SREBP-2 regulates the transcription of LDLR for cellular uptake of LDL cholesterol. At a high concentration of sterol, the precursor SREBPs (pre-SREBPs) become complex with SCAP and Insig proteins and are retained in the endoplasmic reticulum (ER) (7, 8). At a low concentration of sterol, Insig proteins are no longer bound to SCAP and the pre-SREBP complex, causing them to translocate to the Golgi apparatus. There, pre-SREBPs undergo proteolytic cleavage by serine proteases, resulting in the liberation of the N-terminal region, which is a nuclear SREBP. Nuclear SREBP enters the nucleus and binds a sterol regulatory element (SRE) in the promoter region of target genes such as LDLR (9–12). SREBP-regulated gene expression has its own negative intrinsic regulatory mechanism.

Dietary soy isoflavones, including genistein, have received great attention as anti-atherogenic foods due to their lipid-improving effects (13–19), but *in vivo* and *in vitro* findings regarding their total and/or LDL cholesterol–lowering effects have been inconsistent (18, 20–25). Some studies report that dietary supplementation with genistein, a strong bioactive soy isoflavone, lowers total and LDL-cholesterol levels (18, 23, 24), while others describe no effect of soy isoflavones on the concentration of LDL cholesterol, even if vascular function is improved (25).

In the current study, we sought to understand the molecular actions of genistein on the expression of LDLR in hepatocytes and implications for observed hypocholesterolemic or anti-atherogenic effects. Although LDLR is expressed in nearly all tissues, liver LDLR plays a pivotal role in the clearance of LDL cholesterol (26).

## Materials and methods

### Cell culture

Human hepatoblastoma (HepG2) cells were purchased from the Korean Cell Line Bank (Seoul, Korea) and grown in 5% CO_2_ at 37°C in high-glucose DMEM medium (Welgene, Daegu, Korea) containing 10% (v/v) FBS (Cellgro, Manassas, VA, USA) and 5% AA (Invitrogen, Carlsbad, CA, USA). For experiments, cells were plated at a density of 1.6×10^6^ cells per 60 mm dish. The next day, the medium was changed to a serum-starvation medium containing 0.5% (v/v) FBS. After overnight incubation, the cells were treated with genistein at a final concentration of 40 µM for the indicated periods of time. For JNK inhibition experiments, cells were pre-treated with the JNK inhibitor SP600126 (Calbiochem, Darmstadt, Germany) at a concentration of 10 µM for 30 min prior to the addition of genistein.

### Plasmids and cloning

A previously described reporter plasmid pSRE-luc was a gift from Dr. Shimano (27). A luciferase reporter gene plasmid containing the LDLR promoter region was constructed. A promoter fragment of the human LDLR gene from −805 to +50 relative to the transcription start site was amplified from genomic DNA extracted from HepG2 cells by PCR using the following primers: forward 5’-AACTCGAGTTGGTCTCCACCAGCTCTCT-3’ and reverse 5’-TGAAGCTTTCACGACCTGCTGTGTCCTA-3’. The PCR product was subcloned into the XhoI and HindIII sites of the pGL3 basic luciferase vector (Promega, Madison, WI, USA) to generate pLDLR-luc (−805 to +50). Using the parental clone (pLDLR-luc (−805 to +50)), a 5’ deletion construct containing the region from −171 to +50 (pLDLR (−171 to +50)) of the human LDLR gene was generated by PCR using the following primers: forward 5’ – AACTCGAGGGACTGGAGTGGGAATCAGA-3’ and reverse 5’-TGAAGCTTTCACGACCTGCTGTGTCCTA-3’. SRE deletion constructs of pLDLRdelSRE-luc (−805 to +50) and pLDLRdelSRE (−171 to +50) were generated using the following primers: forward 5’-TGAAGACATTTGAAATGCAAACTCCTCCCCCTGCT-3’ and reverse 5’-GGGGAGGAGTTTGCATTTCAAATGTCTTCACCTCACTGC-3’.

### Transient transfection and luciferase assay

HepG2 cells were seeded in 6-well plates (Corning Costar Corp., Tewksbury, MA, USA) at 1.6×10^5^/well, 24 h prior to transfection. Transfections were performed with 0.5 µg of each DNA construct and pRLSV40 (Promega, Madison, WI, USA) using Lipofectamin2000 (Life Technologies, Inc., Grand Island, CA, USA) according to the manufacturer's instructions. Twenty-four hours later, genistein was added to the medium. The next day, the cells were washed three times in PBS and lysed with 100 µL of passive lysis buffer. The clear cell lysate was used for the measurement of luciferase activity using the Dual-luciferase assay system (Promega, Madison, WI, USA) and a Promega Glomax luminometer (Promega BioSystems Sunnyvale Inc., Sunnyvale, CA, USA).

### RNA extraction and quantitative RT-PCR

Trizol reagent (Invitrogen, Carlsbad, CA, USA) was used to extract total RNA from samples, according to the manufacturer's protocol. cDNA was synthesized from 1 µg of total RNA after being primed by random hexamers through reverse-transcription by ImProm-II Reverse Transcriptase (Promega, Madison, WI, USA), according to the manufacturer's directions. Quantitative PCR was performed on a CFX96 sequence detection system (Biorad, Hercules, CA, USA) using EvaGreen qPCR mix plus (Solis BioDyne, Estonia). The levels of expression were normalized relative to the amount of 18S rRNA. Relative mRNA levels were calculated by differences in *C*_*t*_ values and are expressed as the fold change.

### Western blot analysis

HepG2 cells were harvested and lysed in a RIPA buffer containing leupeptin, 1 mM PMSF, Na_3_VO_4_, and 0.1% protease inhibitor cocktails (Sigma, St. Louis, MO, USA). After 30-min incubation on ice, the cells were centrifuged at 13,000 rpm for 20 min at 4°C. The supernatant was mixed with a 5× sample buffer and then boiled for 10 min. Equal amounts of protein cell lysates were loaded onto an SDS-polyacrylamide gel for electrophoresis. PVDF membrane (Millipore, Billerica, MA, USA) was used to transfer proteins from the gel. Mouse anti-SREBP-2 (Santa Cruz Biotech., Santa Cruz, CA, USA), rabbit anti-LDLR (BioVision, Milpitas, CA, USA), rabbit anti-p-JNK (Merck Millipore, Billerica, MA, USA), rabbit anti-p-p38 (Merck Millipore, Billerica, MA, USA), mouse anti-p-ERK (Santa Cruz Biotech., Santa Cruz, CA, USA), and rabbit anti-GAPDH (Signalway antibody, Pearland, TX, USA) antibodies were used to detect the proteins.

### Chromatin immunoprecipitation assay

Cells were crosslinked with ice-cold 1% formaldehyde (Sigma-Aldrich, MO, USA) for 15 min at room temperature. The reaction was stopped by adding 2.5 M of glycine with a final concentration of 125 mM and incubating for 5 min at room temperature. After being rinsed with cold PBS twice, the cells were lysed with 250 µL RIPA buffer (150 mM NaCl, 1% NP-40, 0.5% sodium deoxycholate, 0.1% SDS, 50 mM Tris (pH 8), 5 mM EDTA). The lysates were sonicated four times, 30 s each time, with a sonicator (Sonifier 250, Branson, CT, USA). After centrifugation at 13,000 rpm for 15 min at 4°C, the clear supernatant was used for immunoprecipitation. The pre-cleared protein lysates were gently rotated with anti-SREBP-2 antibody (Santa Cruz Biotech., Santa Cruz, CA, USA) overnight at 4°C and then rotated with a 50% slurry of blocked protein A/G sepharose beads (Santa Cruz, CA, USA) for 2 h at 4°C. Samples were washed with RIPA buffer two times, IP buffer (100 mM Tris (pH 8), 500 mM LiCl, 1% NP-40, 1% sodium deoxycholate) four times, RIPA buffer two times, and TE buffer two times. Every wash process was carried out for 5 min at 4°C. IP material was eluted by adding 200 µL of elution buffer (70 mM Tris (pH 8), 1 mM EDTA, 1.5% SDS) in the remaining 100 µL of TE buffer (10 mM Tris, 1 mM EDTA, pH 7.5) from the last wash step and incubating in a 65°C water bath for 10 min. Chromatin was reverse-crosslinked by adding 13 µL of 4M NaCl to 237 µL of the sample and incubating in a 65°C water bath for 5 h. DNA fragments were purified using a DNA extraction kit (iNtRON Biotechnology, Korea) and PCR was performed with the following primers: LDLR-SRE forward, 5’-TCCTCTTGCAGTGAGGTGAA-3’, reverse, 5’-TTTCTAGCAGGGGGAGGAGT-3’. The PCR generated a 66-bp fragment containing a SRE of the human LDLR promoter.

### Cholesterol uptake assay

HepG2 cells were seeded in 96-well plates (Corning Costar Corp., Tewksbury, MA, USA) at 1×10^4^/well and 24 h later were treated with 40 µM genistein. Cell then equilibrated with 10 µg/mL NBD-labeled cholesterol (Cayman Chemical Company, Ann Arbor, MI, USA) for an additional 12 h. Cells exposed to NBD-labeled cholesterol were washed with PBS and incubated in DMEM for 12 h. Fluorescence-labeled cholesterol was detected by using a fluorometer (TECAN Infinite 200, Switzerland) with excitation and emission of 485 nm and 535 nm, respectively.

### Statistical analyses

SPSS was used to perform statistical analyses (Statistical Package for the Social Sciences; SPSS Inc., Chicago, IL, USA). One-way analysis of variance (ANOVA) followed by Duncan's multiple comparisons test was used to determine statistically significant differences among the experimental groups. *P* values <0.05 were considered statistically significant.

## Results

### Genistein increases LDLR expression

**Fig. 1:**
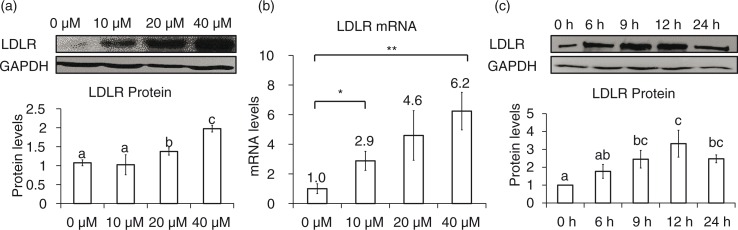
Genistein stimulation of LDLR expression at the transcriptional level. HepG2 cells were serum-starved and then stimulated with genistein. Protein (a) and mRNA levels (b) of LDLR in response to 0, 10, 20, and 40 µM of genistein for 24 h, changes in protein levels (c) of LDLR after incubation with 40 µM of genistein within 24 h. Results are expressed as mean±SD of at least three independent experiments. Different letters (a–c) represent significant differences (*p*<0.05) between all groups by ANOVA or two groups by student's *t*-test. *p<0.05, **p<0.01.

**Fig. 2:**
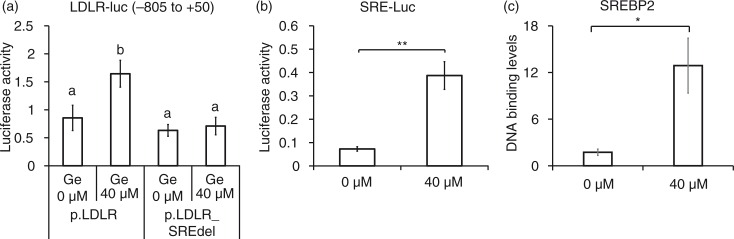
SRE-dependent effects of genistein on LDLR promoter activity. Luciferase activities of pLDLR-luc (−805 to +50) and its corresponding SRE deletion construct (a) and pSRE-luc construct (b) upon the treatment of 40 µM genistein and DMSO (G0 µM) for 24 h. CHIP assay for binding of SREBP-2 to SRE regions of the human LDLR promoter region (c). The results are expressed as mean±SD of at least three independent experiments. Different letters (a–c) represent significant differences (*p*<0.05) for the comparison of all groups by ANOVA (a) or two groups by student's *t*-test (b and c). *p<0.05, **p<0.01.

LDLR protein expression was examined in HepG2 cells after incubation with varying concentrations of genistein. As the concentration increased from 10 to 40 µM, the mRNA and protein levels of LDLR increased up to six-fold and two-fold, respectively (*p*<0.05) (Fig. 1a and b). In time course experiments, a significant increase in LDLR protein levels was detected approximately 6 h after the addition of 40 µM genistein (Fig. 1c). Furthermore, when LDLR promoter activity was investigated using a luciferase construct containing the proximal region of the LDLR gene, genistein increased the transcriptional activity of the construct (Fig. 2a). These results suggest that genistein increases the expression of LDLR at the transcriptional level.

### Genistein-induced expression of LDLR requires SREBP-2 transcriptional activity

We investigated whether SREBP-2 is involved in the genistein-mediated upregulation of LDLR, based on the fact that SREBP-2 is a crucial transcription factor for the LDLR gene. In order to examine whether SREBP-2 transcriptional activity is required for genistein-mediated upregulation of the LDLR gene, luciferase reporter constructs were generated. Genistein significantly increased luciferase activity, and this effect was counteracted by an SRE deletion construct (Fig. 2a). The luciferase construct driven by SRE alone, which originated from the human SREBP-2 promoter region, responded to genistein by increasing luciferase activity (Fig. 2b). In addition, the DNA-binding activity of SREBP-2 upon genistein treatment was higher, as tested by chromatin immunoprecipitation assay (CHIP) assay (Fig. 2c). These results clearly suggest that SREBP-2 transcriptional activity is required for the genistein-driven upregulation of the LDLR gene.

### Genistein activates the JNK signaling pathway for SREBP-2 processing

**Fig. 3:**
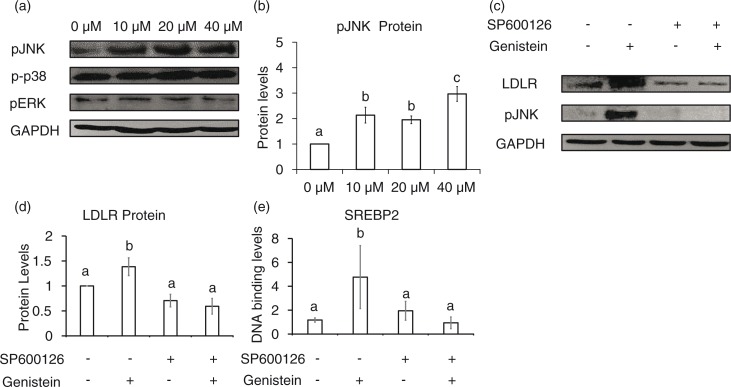
JNK activation by genistein involves SREBP-2 binding activity and LDLR expression. Phosphorylated levels of JNK, p38, and ERK upon 10, 20, or 40 µM genistein treatment for 24 h (a) and quantification of p-JNK protein levels (b). LDLR and p-JNK protein levels with pre-treatment of SP600126 or vehicle DMSO (c) and quantification of LDLR protein levels (d). CHIP assay for binding of SREBP-2 with pre-treatment of SP600126 or vehicle DMSO (e). The results are expressed as mean±SE of at least three independent experiments. Different letters (a–c) represent significant differences between all groups (*p*<0.05) by ANOVA.

Next, we asked whether mitogen-activated protein kinase (MAPK) signaling pathways are involved in genistein-mediated LDLR expression. Genistein did not significantly alter the levels of the phosphorylated form of p38 and pERK proteins (Fig. 3a), but phosphorylation of JNK protein increased in a concentration-dependent manner (Fig. 3a and b). To assess whether JNK is involved in the regulation of the LDLR gene by genistein, we treated cells with a JNK-specific inhibitor, SP600126, which greatly reduced the phosphorylation of JNK and LDLR protein levels that had been increased by genistein (Fig. 3c and d). In addition, the DNA-binding activity of SREBP-2 was significantly decreased upon addition of SP600126 (Fig. 3e). These results indicate that the activation of JNK signals is necessary for genistein-induced LDLR expression.

### Genistein did not upregulate SREBP-1c mRNAs

**Fig. 4:**
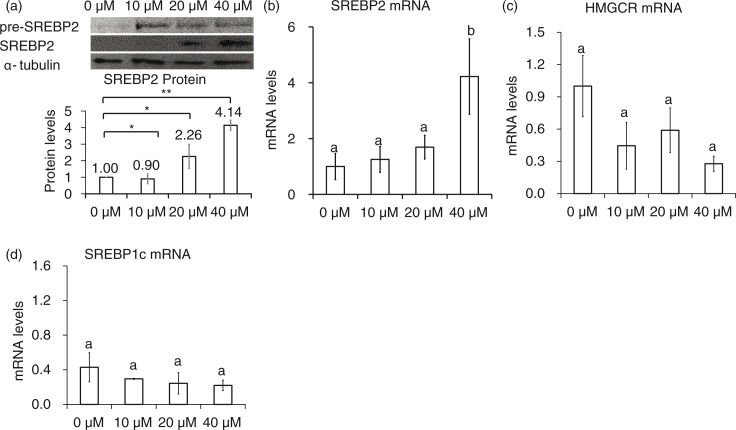
Nuclear SREBP-2 and HMGCR levels in genistein-treated cells. Protein (a) and mRNA levels of SREBP-2 (b), and HMGCR (c) in cells treated with 10, 20, and 40 µM genistein for 24 h. The results are expressed as mean±SE of at least three independent experiments. Different letters (a–c) represent significant differences between all groups (*p*<0.05) by ANOVA or two groups by student's *t*-test. *p<0.05, **p<0.01.

Genistein significantly upregulated mature SREBP-2 protein after the addition of 20 to 40 µM for 24 h (Fig. 4a), but the mRNA levels of SREBP-2 was greatly elevated only after the addition 40 µM genistein (Fig. 4b). Although genistein markedly increased LDLR mRNA levels, 24-h incubation with genistein did not significantly upregulate HMGCR mRNA levels (Fig. 4c). SREBP-1c did not change its expression levels in response to genistein (data not shown). Together, it is likely that there are other regulatory factors affecting the expression levels of HMGCR and SREBP-1c.

### Cholesterol uptake increased with genistein treatment

**Fig. 5:**
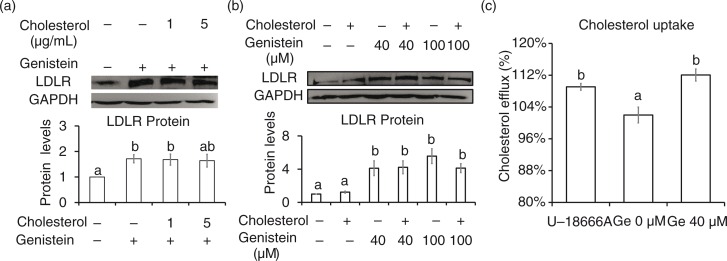
Cholesterol did not affect Genistein ability to elevate LDLR expression. Protein levels of LDLR and GAPDH in cells incubated with 40 (a) or 100 µM (b) of genistein in the presence of either cholesterol (1 and 5 µg/mL) or solvent alone (0.1% ethanol) for 24 h. Enhancement of cholesterol uptake in cells treated with genistein 40 µM for 24 h. The results are expressed as mean±SE of at least three independent experiments. Different letters (a–c) represent significant differences between all groups (*p*<0.05) by ANOVA.

Then we asked whether cholesterol affects the regulation of genistein on SREBP-2 processing and LDLR expression. The addition of cholesterol with concentration from 1 to 5 µg/mL did not lower LDLR protein levels (Fig. 5a and b). Even the cholesterol uptake were increased from 101 to 112% with the treatment of 40 µM genistein (Fig. 5c). These data suggest that the elevation in intracellular cholesterol concentration did not affect the ability of genistein to promote LDLR protein.

## Discussion

In a previous animal study, the anti-atherogenic effects of genistein appeared to mediate LDLR based on a lack of the effects in LDLR knock-out mice (17), but the exact molecular mechanism behind the actions of genistein was not investigated. Here, we demonstrate that genistein activates the JNK signaling pathway and increases SREBP-2 processing for the SRE-dependent transcription of the LDLR gene in human hepatocytes. Because hepatic LDLR plays a crucial role in the regulation of blood LDL cholesterol, the upregulation of LDLR in hepatocytes by genistein is likely to contribute to its anti-atherogenic effects.

LDLR is regulated by various cellular stimuli such as growth factors, insulin (28), and estradiol (28, 29). SRE is required for the insulin- or estradiol-induced expression of the LDLR gene (28). The maturation of SREBP by proteolytic cleavage has been implicated in regulation by growth factors such as platelet-derived growth factor (30). Our data showed that genistein also upregulates LDLR expression at transcriptional levels, which requires SRE-mediated transcriptional activity of SREBP-2. The genistein-induced transcriptional activity of SREBP-2 appears to be due to an increase in the amount of nuclear SREBP-2.

Intracellular signaling mechanisms involved in the soy isoflavone–mediated upregulation of the LDLR gene have not been extensively elucidated. MAP kinase–related signals have been implicated in the transcriptional activation of SREBPs (31). Here, we provide evidence of the role of JNK activation on SREBP-2 processing and LDLR expression. JNK inhibition greatly reduced genistein-increased LDLR expression, suggesting that the JNK pathway links the effects of genistein and the activation of SREBP-2 for LDLR expression. The activation of JNK signaling pathway has been known as a key modulator in apoptosis (32) and cell death (33). However, the addition of 40 µM of genistein did not interfere with cell viability in HepG2 cells (34) as well as in the other types of cells (35, 36). In this case, the activation of JNK signaling was the only elevated LDLR expression without affected cell viability. The involvement of JNK activation in LDLR expression has been previously reported in berberine-treated cells (37). However, the effect of berberine on JNK activation and LDLR expression is SRE-independent and mediated by the transcriptional activity of c-jun, which is phosphorylated upon JNK activation (37). JNK activation is better understood in the regulation of SREBP-1c-mediated gene expression. JNK increases insulin-mediated activation of SREBP-1c for *de novo* fatty acid synthesis (31). Another MAPK, ERK, mediates the signals initiated by insulin and platelet-derived growth factor (38). ERKs directly phosphorylate nuclear SREBPs and lower the recruitment of SUMO-mediated HDAC, resulting in the enhancement of SREBP transcriptional activity (39).

SREBP-2 activation by genistein could result in the expression of other target genes, such as HMGCR and SREBP-2 itself. We observed that HMGCR mRNA levels were not significantly upregulated by genistein, which may indicate that factors other than SREBP-2, as affected by genistein, influence the regulation of HMGCR and LDLR expression. SREBP-2 mRNA levels were elevated after the addition of genistein 40 µM for 24 h, suggesting that a positive feedback loop of SREBP-2 enhanced SREBP-2 mRNA production. The effect of genistein treatment was clearly shown in the protein SREBP-2 in mature form when a nuclear extraction compared with mRNA levels of SREBP-2 was performed. On the contrary, SREBP-1c, which is under the control of sterol-regulated processing similar to that of SREBP-2, was not significantly upregulated by genistein (data not shown).

LDLR expression is controlled in a tight negative feedback mechanism by intracellular sterol levels (26). The LDLR-lowering effects of genistein were not sensitive to the presence of cholesterol. Our data showed that cholesterol did not affect genistein-induced SREBP-2 processing and LDLR expression, even though our result showed that cholesterol uptake in genistein treatment was increased. Besides the elevation of LDLR, genistein may also elevate the uptake of cholesterol by increasing the expression of SR-BI, as shown in the HepG2 cells that were treated with estrogen (40) or cacao polyphenols (41). A previous study (40) proved that genistein inhibited cellular cholesterol synthesis and decreased cellular cholesterol esterification, and the increase of cholesterol uptake by genistein enriched our knowledge about the ability of genistein to play an important protective role in the development of atherosclerosis.

## Conclusion

In conclusion, our results demonstrate that genistein, a soy isoflavone, could act as an LDL-cholesterol-lowering agent via JNK activation and SREBP-2 processing in hepatocytes. Our finding broadens the understanding of how genistein induces LDLR expression and increases cholesterol uptake, and will guide the development of genistein as an anti-hypocholesterolemic.
